# MRPL12 Acts as A Novel Prognostic Biomarker Involved in Immune Cell Infiltration and Tumor Progression of Lung Adenocarcinoma

**DOI:** 10.3390/ijms24032762

**Published:** 2023-02-01

**Authors:** Yangyang Hu, Yue Liu, Chenchao Ma, Kaixing Ai

**Affiliations:** Department of Thoracic Surgery, Shanghai Pulmonary Hospital, School of Medicine, Tongji University, Shanghai 200433, China

**Keywords:** MRPL12, LUAD, prognosis, immune infiltration, biomarker

## Abstract

Mitochondrial ribosomal protein L7/L12 (MRPL12) is a member of the mitochondrial ribosomal proteins (MRPs). However, the biological function of MRPL12 in lung adenocarcinoma (LUAD) remains unclear. The expression and prognostic value of MRPL12 in LUAD were systematically analyzed using UALCAN, TIMER, HPA, Kaplan–Meier plotter, and GEPIA databases. The relationship between MRPL12 and immune infiltrates was investigated using TIMER and TISIDB databases. The clinical significance of MRPL12 in LUAD patients was validated using a tissue microarray (TMA). Cellular functional experiments were carried out to examine the influences of MRPL12 knockdown on cell proliferation, migration, and invasion. MRPL12 was significantly upregulated in LUAD samples, and high MRPL12 expression was correlated with worse prognosis. MRPL12 expression was markedly associated with immunomodulators, chemokines, and infiltration levels of multiple immune cells. Furthermore, TMA results confirm the upregulation of MRPL12 expression in LUAD, and MRPL12 was identified as an independent prognostic factor in LUAD patients. MRPL12 knockdown inhibited proliferation, migration, and invasion of LUAD cells. These data indicate that MRPL12 is a prognostic biomarker and correlated with immune infiltrates in LUAD. Therefore, MRPL12 shows potential as a therapeutic target for LUAD.

## 1. Introduction

Lung cancer is the second most frequent malignancy and the leading cause of cancer-related deaths worldwide [1]. Non-small-cell lung cancer (NSCLC) accounts for around 85% of lung cancer cases, with lung adenocarcinoma (LUAD) being the most common histological subtype [2]. Despite great improvements in therapeutic strategies in recent years, the prognosis of LUAD patients remains poor [3]. The 5-year survival rate of patients with LUAD was reported to be less than 20% [4]. Therefore, studies are urgently needed exploring novel biomarkers and therapeutic targets to improve the survival of LUAD patients.

Mitochondrial ribosomal protein L7/L12 (MRPL12) is one of the mitochondrial ribosomal proteins (MRPs), which are indispensable for the structural and functional integrity of the mitochondrial ribosome complex [5]. In addition, MRPL12 interacts with mitochondrial RNA polymerase to directly activate transcription in the mitochondria [6]. Mitochondria play pivotal roles in the cellular processes and functions of malignancies, and accumulating evidence strongly indicates that alteration in MRP levels is correlated with tumor development [7]. For example, Min et al. found MRPS31 deficiency acts as an important driver in mitochondrial dysfunction in hepatocellular carcinoma, and MRPS31 suppression significantly promotes hepatoma cell invasion [8]. MRPL12 exerts a pathogenic role in several diseases, including diabetic kidney disease and cancer [9,10]. Liu et al. demonstrated that upregulation of MRPL12 served as an independent risk factor and correlated with poor survival of breast cancer patients; MRPL12 knockdown markedly inhibited cell viability and migration in breast cancer [11]. Nevertheless, the biological function of MRPL12 in LUAD remains unknown.

Tumor-infiltrating immune cells (TIICs) are a key component of the tumor microenvironment (TME) and play a critical role in the initiation and development of malignancies [12]. Moreover, the type and density of TIICs in the TME affect the survival outcomes of cancer patients; for example, reduced CD4+ central memory T cells and natural killer (NK) cells were significantly associated with better survival in glioblastoma patients [13]. B cells and myeloid dendritic cells (DCs) were identified as independent prognostic factors for lung cancer patients, and further enrichment analyses reveled that genes highly related to B cells or myeloid DCs were closely related to immune activation in these patients [14]. Therefore, investigating targets linked to immune infiltration is important for LUAD therapy. However, associations between MRPs and immune infiltration patterns have not been widely examined. MRPL15 expression was markedly upregulated and associated with immune infiltration in NSCLC, including in NK cells, mast cells, immature B cells, and eosinophils [15]. The potential association between MRPL12 and TIICs in LUAD is unclear.

In this research, the TIMER and UALCAN databases were utilized to assess MRPL12 mRNA expression in LUAD. The MRPL12 protein level was analyzed using the Human Protein Atlas (HPA) database, and verified using tissue microarray (TMA) samples. GEPIA and the Kaplan–Meier plotter database were applied to analyze how MRPL12 affects the overall survival (OS) of LUAD patients. The correlation between MRPL12 expression and TIICs in LUAD was investigated within the TIMER and TISIDB databases. The relationship between MRPL12 expression and gene markers of TIICs was evaluated using the TIMER and GEPIA databases. GeneMANIA and LinkedOmics analyses were used to further explore genes co-expressed with MRPL12 and their regulatory networks. To confirm the functions of MRPL12 in cell proliferation, migration, and invasion, several cellular experiments were conducted. This study provides insight into the prognosis as well as immune-infiltration-related role of MRPL12 in LUAD. 

## 2. Results

### 2.1. MRPL12 Expression Level in LUAD

**Figure 1:**
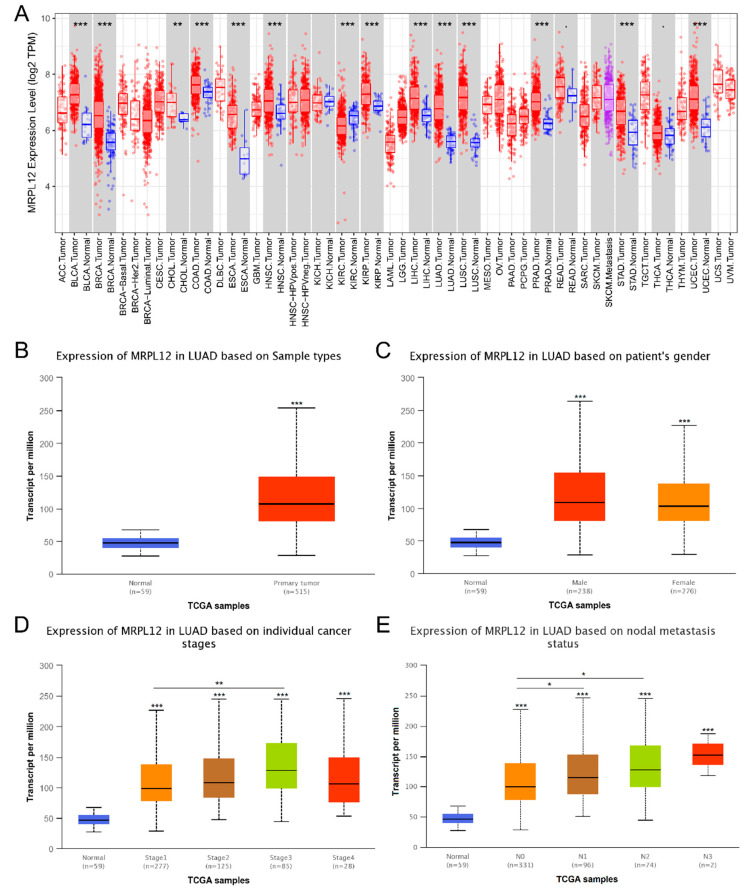
MRPL12 mRNA expression level in LUAD. (**A**) MRPL12 mRNA level was upregulated in various types of malignancies from TIMER database, including LUAD. (**B**) MRPL12 mRNA level was markedly increased in LUAD tissues from UALCAN database. (**C**–**E**) MRPL12 mRNA level was overexpressed in LUAD samples compared with normal samples, as analyzed by patients’ gender, cancer stage, and nodal metastasis status. Multiple group comparisons were performed with ANOVA followed by the Bonferroni post hoc test. * *p* < 0.05, ** *p* < 0.01, *** *p* < 0.001.

Gene expression analyses in the TIMER database revealed that MRPL12 mRNA expression was significantly overexpressed in multiple tumor tissues in comparison to corresponding normal tissues, including LUAD (Figure 1A). MRPL12 expression in LUAD was validated using the UALCAN database, and MRPL12 was also upregulated in LUAD samples (Figure 1B). Subgroup analyses of several pathological parameters of LUAD patients were performed using the UALCAN database. MRPL12 mRNA expression was higher in LUAD tissues than in normal tissues and analyzed by gender (Figure 1C), cancer stages (Figure 1D), and nodal metastasis status (Figure 1E). 

**Figure 2:**
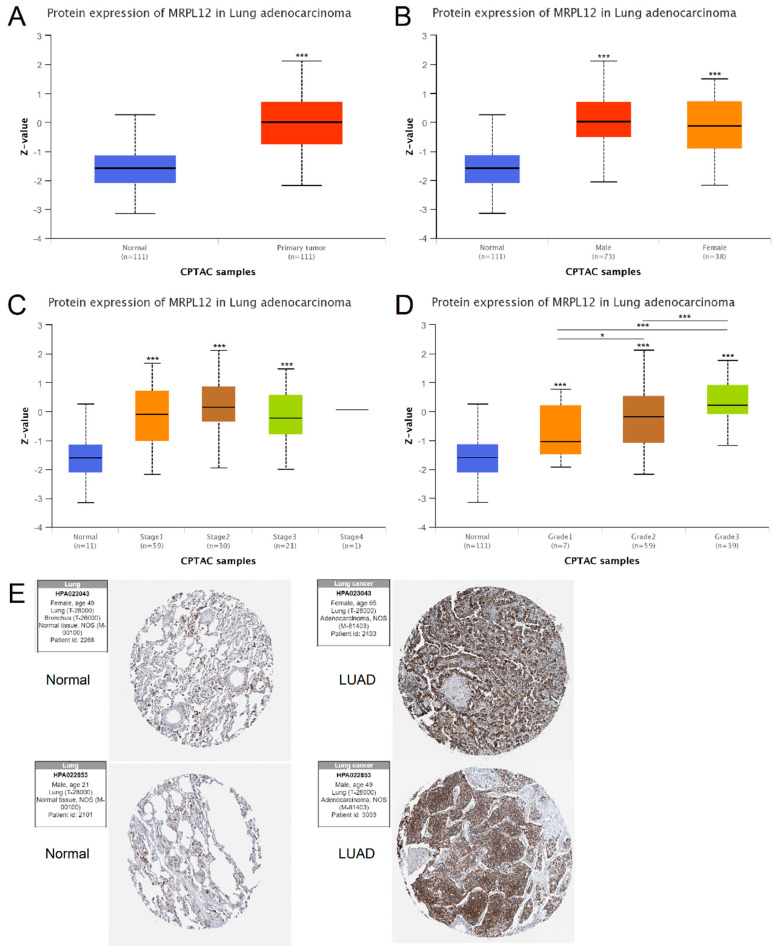
MRPL12 protein expression level in LUAD. (**A**) MRPL12 protein level was upregulated in LUAD tissues from UALCAN. (**B**–**D**) MRPL12 protein level was increased in LUAD samples compared with normal samples and analyzed by gender, cancer stages, and tumor grade. (**E**) IHC images of MRPL12 in LUAD and normal lung tissues in the HPA database. Multiple group comparisons were performed with ANOVA followed by the Bonferroni post hoc test. * *p* < 0.05, *** *p* < 0.001.

MRPL12 protein expression was then analyzed in the UALCAN database. As presented in Figure 2A, MRPL12 protein was obviously upregulated in LUAD samples in comparison to normal samples. Further subgroup analyses of gender, cancer stages, and tumor grade clearly revealed that the protein expression of MRPL12 was higher in LUAD tissues than in normal tissues (Figure 2B–D). Moreover, immunohistochemistry (IHC) analysis in the HPA database showed that MRPL12 was overexpressed in LUAD tissues compared with normal lung tissues (Figure 2E). These findings indicate that MRPL12 functions as a diagnostic biomarker in LUAD, regardless of the different pathological characteristics of patients.

### 2.2. Prognostic Value of MRPL12 in LUAD

**Figure 3:**
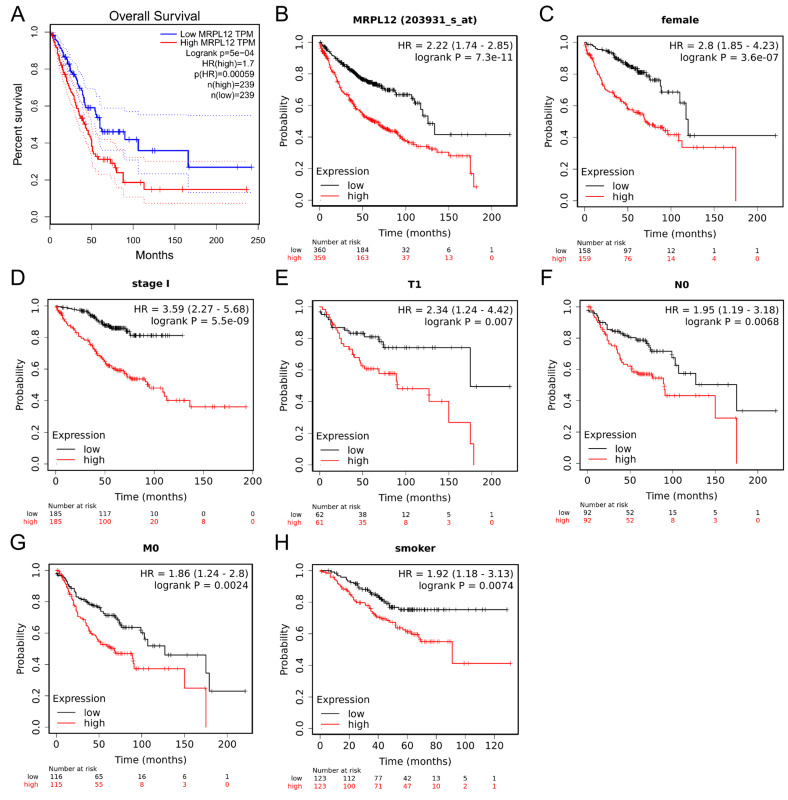
Prognostic value of MRPL12 in LUAD. (**A**,**B**) High MRPL12 expression was related to shorter OS in LUAD patients from GEPIA and Kaplan–Meier plotter databases. (**C**–**H**) High MRPL12 expression was related to worse OS in different subgroups, including female, stage I, T1, N0, and M0, and smoker cohorts.

A Kaplan–Meier survival curve was applied to evaluate the correlation between MRPL12 expression and the OS of LUAD patients. Analysis using the GEPIA database, which was based on TCGA, revealed that high MRPL12 expression was related to a worse OS in LUAD patients (Figure 3A). The prognostic value of MRPL12 was verified in the Kaplan–Meier plotter database. Increased MRPL12 expression was consistently associated with a shorter OS in the LUAD cohort (Figure 3B). Next, the relationship between MRPL12 expression and OS in different subgroups was explored. LUAD patients with high MRPL12 expression suffered a worse OS in multiple subgroups, including female, stage I, T1, N0, and M0, and smoker cohorts (Figure 3C–H). These results suggest that MRPL12 can be used as a biomarker for predicting the prognosis of LUAD patients.

### 2.3. Correlation between MRPL12 Expression and Survival of LUAD Patients in TMA Cohort

**Figure 4:**
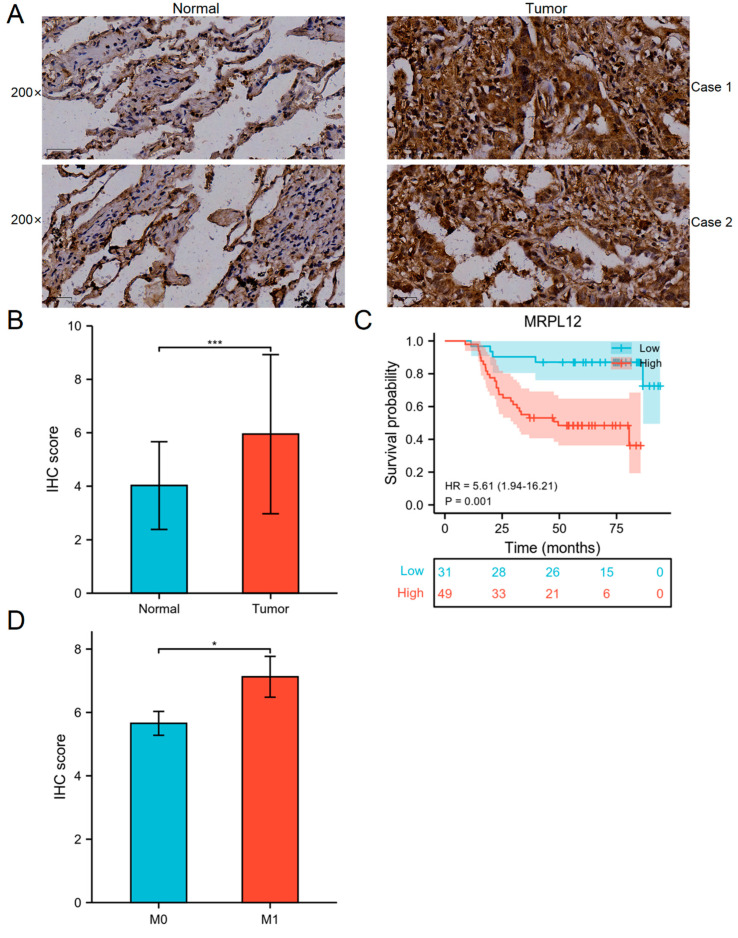
Correlation between MRPL12 expression and prognosis of LUAD patients from TMA. (**A**) IHC images of MRPL12 in LUAD and matched normal tissues. (**B**) IHC score of MRPL12 was higher in LUAD tissues. (**C**) High MRPL12 expression in LUAD patients was related to worse survival. (**D**) IHC score of patients with metastasis was higher than that of patients without metastasis. * *p* < 0.05, *** *p* < 0.001.

The protein expression and prognostic value of MRPL12 in LUAD patients were validated using the TMA, which comprises 80 paired LUAD and matched normal tissues. The clinicopathological information of these patients is shown in Table 1. MRPL12 expression was remarkably associated with pathologic stage (*p* = 0.003). IHC analysis revealed that MRPL12 was overexpressed in LUAD tissues compared to normal tissues (Figure 4A). Additionally, the IHC score of MRPL12 was higher in LUAD tissues (Figure 4B).

Survival analysis indicated that high MRPL12 expression was significantly associated with a worse OS (Figure 4C), which is consistent with TCGA data. The IHC score of patients with metastasis was higher than that of patients without metastasis (Figure 4D). We further identified the prognostic factors for LUAD patients through univariate and multivariate Cox regression analyses. As shown in Table 2, tumor size, T stage, N stage, pathological stage, chemotherapy, and MRPL12 expression were correlated with the OS of LUAD patients according to the univariate analysis. These significant variables were included in the multivariate analysis, and T stage, pathological stage, and MRPL12 expression were independent prognostic factors (Table 2).

### 2.4. GO and KEGG Pathway Enrichment Analysis of MRPL12

**Figure 5:**
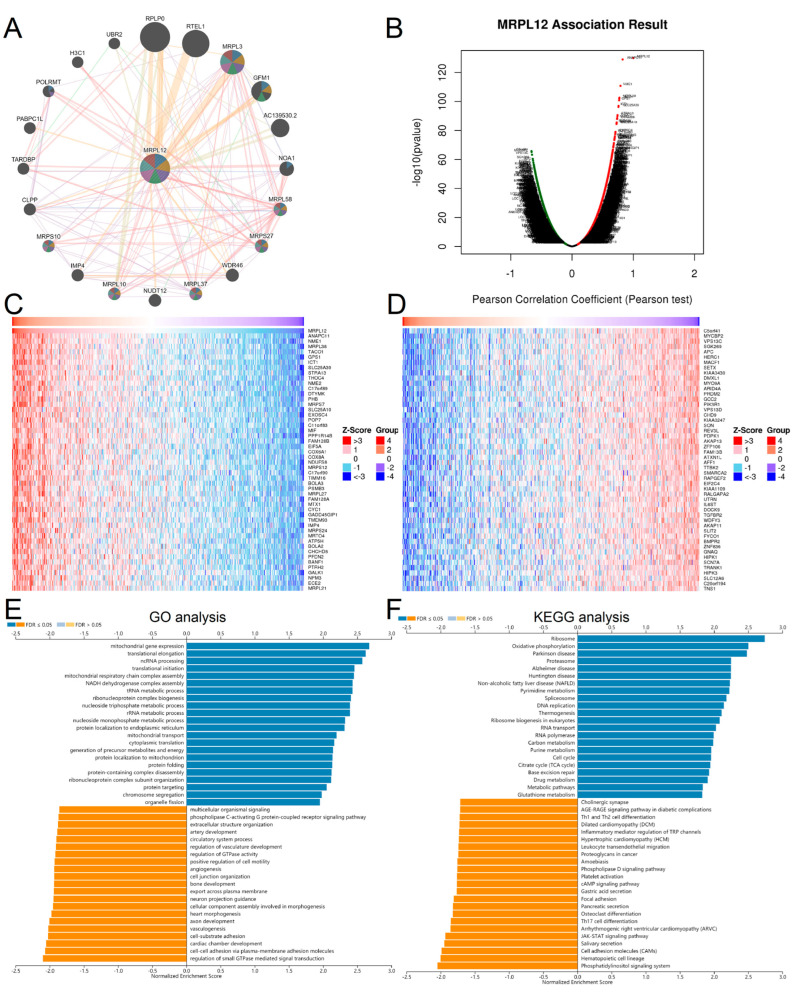
GO and KEGG pathway enrichment analysis of MRPL12 in LUAD. (**A**) PPI network of MRPL12 with its interactive genes from GeneMANIA database. (**B**) Volcano plot of all genes correlated with MRPL12 in LUAD from LinkedOmics database. (**C**,**D**) Heatmaps of top 50 significant genes positively and negatively correlated with MRPL12. (**E**) GO analysis of MRPL12 in LUAD. (**F**) KEGG pathway analysis of MRPL12 in LUAD.

The protein–protein interaction (PPI) network of MRPL12 was investigated with GeneMANIA (Figure 5A). The top 10 proteins predicted to interact with MRPL12 were RPLP0, RTEL1, MRPL3, GFM1, AC139530.2, NOA1, MRPL58, MRPS27, WDR46, and MRPL37. 

To investigate the potential biological functions of MRPL12 in LUAD, we identified its co-expressed genes and related pathways using LinkedOmics. The results show that 19,988 genes were correlated with MRPL12 in LUAD, including 8664 positively and 11,324 negatively correlated genes (Figure 5B). Heatmaps of the top 50 significant genes with positive and negative correlations with MRPL12 are exhibited in Figure 5C,D. Further Gene Ontology (GO) and Kyoto Encyclopedia of Genes and Genomes (KEGG) pathway enrichment analyses were conducted. GO analysis indicated MRPL12 and co-expressed genes participated in mitochondrial gene expression, translational initiation, protein folding, regulation of small-GTPase-mediated signal transduction, cell–substrate adhesion and vasculogenesis (Figure 5E). KEGG pathway enrichment analysis revealed MRPL12 and co-expressed genes participated in ribosomes, oxidative phosphorylation, proteasomes, Th17 cell differentiation, Th1 and Th2 cell differentiation, and the JAK-STAT signaling pathway (Figure 5F).

### 2.5. MRPL12 Expression in TME at the Single-Cell Level

**Figure 6:**
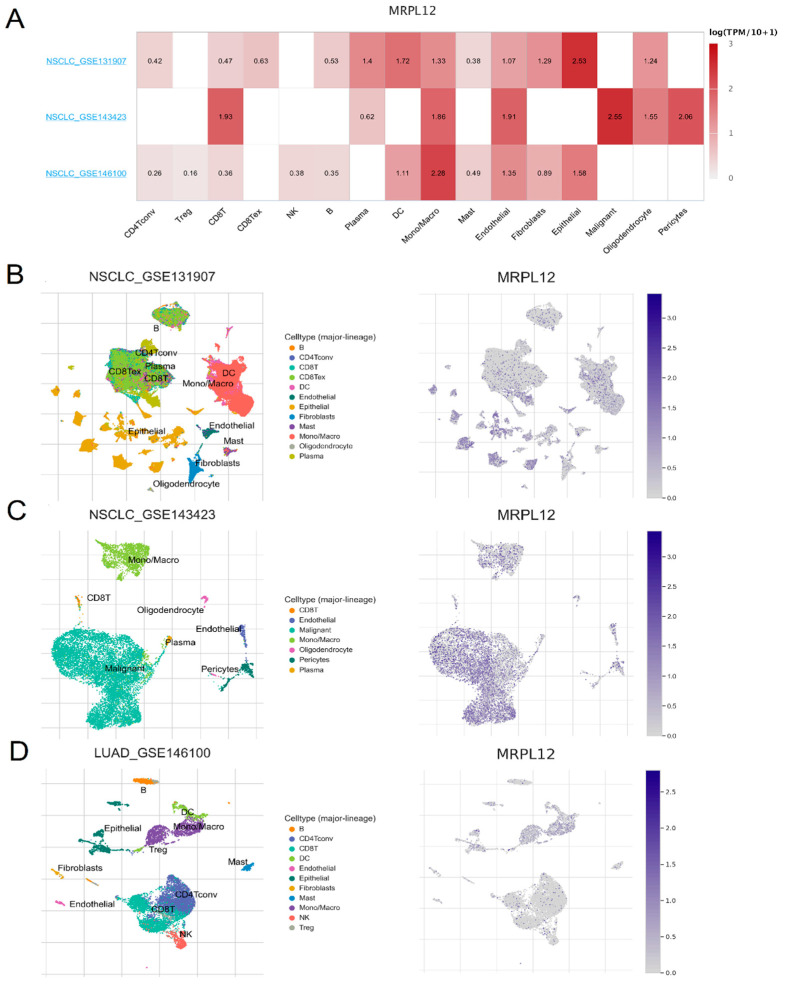
MRPL12 expression in the TME of LUAD at the single-cell level. (**A**) Heatmap of MRPL12 expression value in different cell types from three datasets in TISCH database. (**B**–**D**) The single-cell cluster maps of MRPL12 distribution in NSCLC_GSE131907, NSCLC_GSE143423, and NSCLC_GSE146100 datasets.

Three datasets (NSCLC_GSE131907, NSCLC_GSE143423, and NSCLC_GSE146100) from the Tumor Immune Single-cell Hub (TISCH) database were utilized to explore MRPL12 expression in the TME of LUAD. The distribution of MRPL12 expression in various immune cells from each dataset is shown in Figure 6A. In the NSCLC_GSE131907 dataset, MRPL12 was mainly expressed in B cells, CD4 conventional T (CD4Tconv) cells, CD8T cells, exhausted CD8T (CD8Tex) cells, and DCs (Figure 6B). In the NSCLC_GSE143423 dataset, MRPL12 was mainly expressed in CD8T cells, endothelial cells, malignant cells, mono/macro cells, and oligodendrocytes (Figure 6C). In the NSCLC_GSE146100 dataset, MRPL12 was mainly expressed in B cells, CD4Tconv cells, CD8T cells, DCs, and endothelial cells (Figure 6D). These data indicate that MRPL12 functions in immune cells and stromal cells in addition to in malignant cells. Moreover, MRPL12 expression varied widely in different cell types, which could lead to TME heterogeneity in LUAD.

### 2.6. Correlation between MRPL12 Expression and Immune Infiltrates 

**Figure 7:**
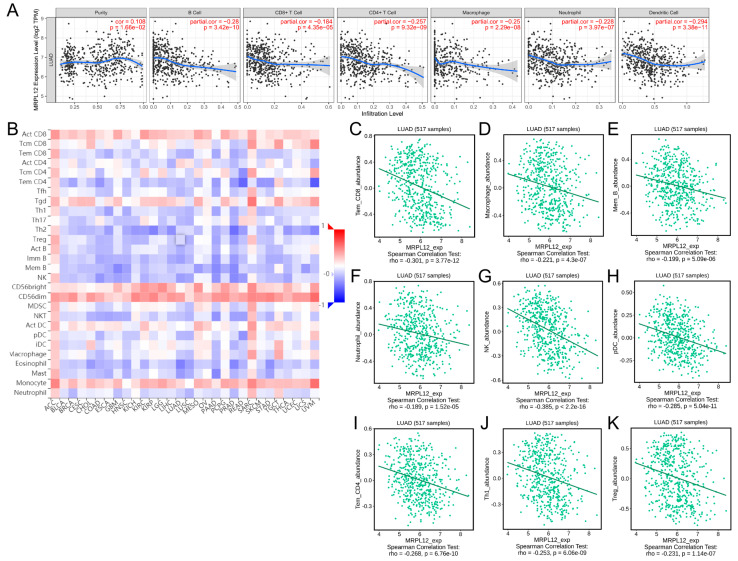
Correlation between MRPL12 expression and immune infiltrates in LUAD. (**A**) Correlation between MRPL12 and abundance of 6 immune cells in TIMER. (**B**) Heatmap of relationship between MRPL12 and abundance of 28 TILs in different tumors using TISIDB database. (**C**–**K**) MRPL12 was negatively related to the abundance of Tem_CD8, macrophages Mem_B, neutrophils, NK cells, pDC, Tem_CD4, Th1 cells, and Tregs in LUAD using TISIDB database.

The association between MRPL12 expression and immune infiltrates was explored using TIMER and TISIDB databases. The data from TIMER revealed that MRPL12 was negatively correlated with the abundance of B cells (r = −0.28, *p* < 0.001), CD8+ T cells (r = −0.184, *p* < 0.001), CD4+ T cells (r = −0.257, *p* < 0.001), macrophages (r = −0.25, *p* < 0.001), neutrophils (r = −0.228, *p* < 0.001), and DCs (r = −0.294, *p* < 0.001) (Figure 7A). In addition, the relationship between MRPL12 expression and the infiltration levels of 28 tumor-infiltrating lymphocytes (TILs) was evaluated via TISIDB. As shown in Figure 7B, MRPL12 was significantly correlated with the abundance of TILs across various cancer types. In LUAD, MRPL12 expression was negatively correlated with the infiltration levels of effector memory CD8 T cells (Tem_CD8, r = −0.301, *p* < 0.001), macrophages (r = −0.221, *p* < 0.001), memory B cells (Mem_B, r = −0.199, *p* < 0.001), neutrophils (r = −0.189, *p* < 0.001), NK cells (r = −0.385, *p* < 0.001), plasmacytoid DCs (pDCs, r = −0.285, *p* < 0.001), effector memory CD4 T cells (Tem_CD4, r = −0.268, *p* < 0.001), Th1 cells (r = −0.253, *p* < 0.001), and Tregs (r = −0.231, *p* < 0.001) (Figure 7C–K). These results indicate MRPL12 is critical for immune cell infiltration of LUAD.

### 2.7. Correlation between MRPL12 Expression and Gene Markers of Immune Cells 

The correlation between MRPL12 expression and different gene markers of immune cells in LUAD was investigated using the TIMER and GEPIA databases. After the adjustment for purity, MRPL12 had a remarkable positive correlation with KIR2DL4 of NK cells, IFNG of Th1 cells, and PDCD1, LAG3, and GZMB of T-cell exhaustion, whereas it exhibited a significant negative correlation with all gene markers of B cells, monocytes, tumor-associated macrophages (TAMs), M2 macrophages, neutrophils, and DCs (Table 3). 

To confirm the above results, we evaluated the relationship between MRPL12 expression and gene markers of T cells, B cells, TAMs, M2 macrophages, DCs, Th1 cells, Th2 cells, and regulatory T cells (Treg) in the GEPIA database. MRPL12 was negatively correlated with the majority of these gene markers (Table 4), which agreed with the results obtained from the TIMER database.

### 2.8. Correlation between MRPL12 Expression and Immunomodulators 

**Figure 8:**
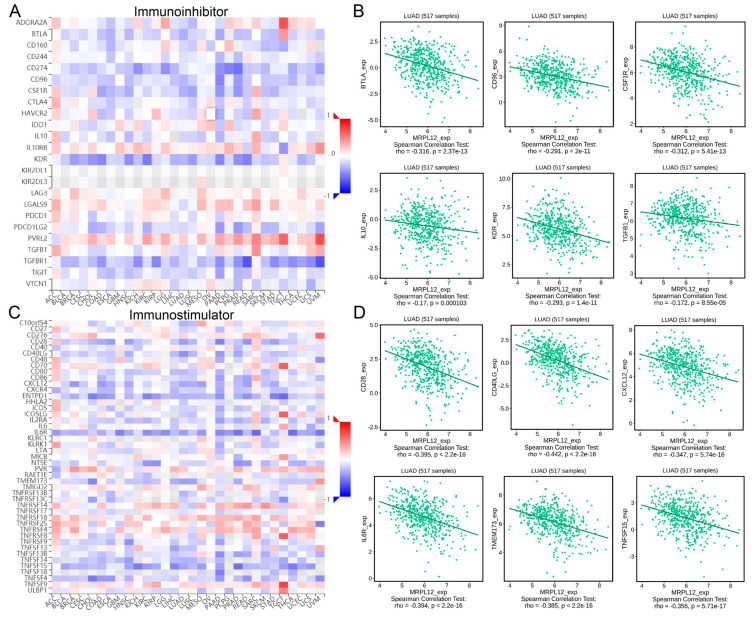
Correlation between MRPL12 expression and immunomodulators in LUAD. (**A**) Heatmap of correlations between MRPL12 and immunoinhibitors across different cancers from TISIDB. (**B**) Relationships between MRPL12 and immunoinhibitors (including BTLA, CD96, CSF1R, IL10, KDR, and TGFB1) in LUAD. (**C**) Heatmap of correlations between MRPL12 and immunostimulators across different cancers. (**D**) Relationships between MRPL12 and immunostimulators (including CD28, CD40LG, CXCL12, IL6R, TMEM173, and TNFSF15) in LUAD.

Immunomodulators, including immunoinhibitors and immunostimulators, are substances that regulate immune system function. Correlations between MRPL12 expression and immunomodulators were explored in TISIDB. A heatmap of the relationships between MRPL12 and immunoinhibitors in different tumors is exhibited in Figure 8A. In LUAD, MRPL12 was negatively correlated with multiple immunoinhibitors, including BTLA, CD96, CSF1R, IL10, KDR, and TGFB1 (Figure 8B). A heatmap of the relationships between MRPL12 and immunostimulators in various tumors is shown in Figure 8C. In LUAD, a negative correlation was observed between MRPL12 and CD28, CD40LG, CXCL12, IL6R, TMEM173, and TNFSF15 (Figure 8D).

### 2.9. Correlation between MRPL12 Expression and Chemokines 

**Figure 9:**
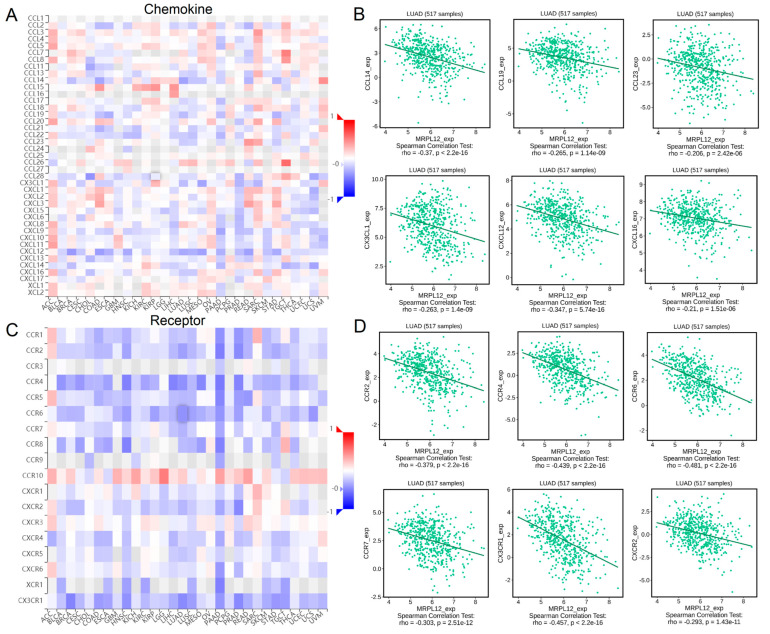
Correlation between MRPL12 expression and chemokines in LUAD. (**A**) Heatmap of correlations between MRPL12 and chemokines in different cancers from TISIDB database. (**B**) Correlations between MRPL12 expression and chemokines (including CCL14, CCL19, CCL23, CX3CL1, CXCL12, and CXCL16) in LUAD from TISIDB database. (**C**) Heatmap of correlations between MRPL12 and receptors across different tumors from TISIDB database. (**D**) Relationships between MRPL12 and receptors (including CCR2, CCR4, CCR6, CCR7, CX3CR1 and CXCR2) in LUAD from TISIDB database.

Chemokines and chemokine receptors play a crucial role in the recruitment and activation of immune cells. Correlations between MRPL12 expression and chemokines were evaluated in TISIDB. MRPL12 was negatively correlated with various chemokines (Figure 9A), including CCL14, CCL19, CCL23, CX3CL1, CXCL12, and CXCL16 (Figure 9B). Meanwhile, MRPL12 was negatively correlated with different chemokine receptors (Figure 9C), including CCR2, CCR4, CCR6, CCR7, CX3CR1, and CXCR2 (Figure 9D). These findings suggest that MRPL12 is an immunoregulatory factor in LUAD.

### 2.10. Prognostic Value of MRPL12 in LUAD Based on Immune Cells 

**Figure 10:**
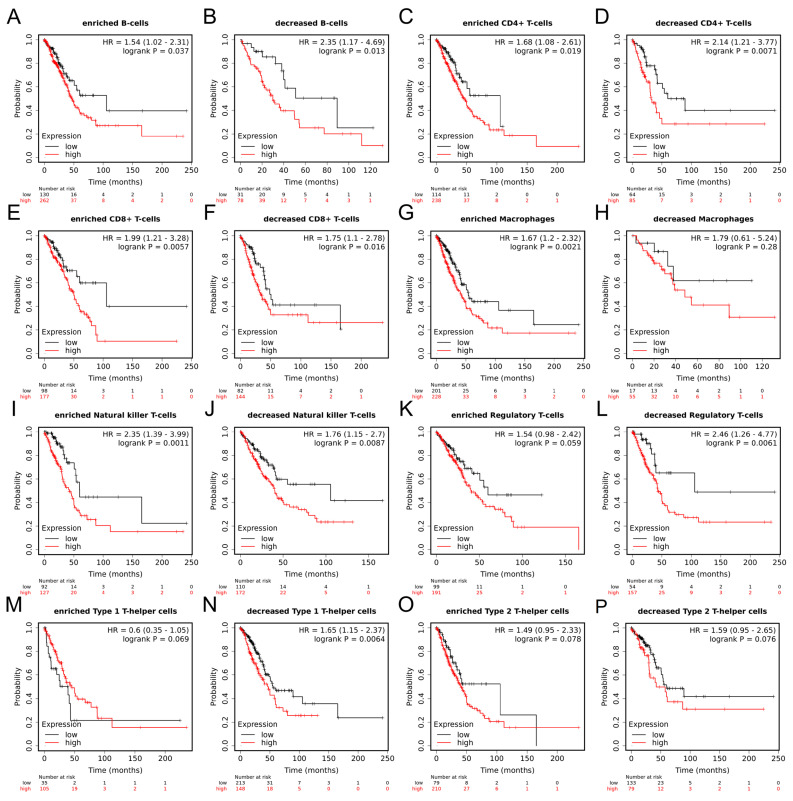
Prognostic value of MRPL12 in LUAD based on immune cell subgroups. (**A**–**P**) The prognostic value of MRPL12 expression in LUAD patients based on various immune cell subgroups using Kaplan–Meier plotter database.

Moreover, survival analysis according to MRPL12 expression in different immune cell subgroups was performed using Kaplan–Meier plotter database. Higher MRPL12 expression was related to a worse OS in enriched and decreased B cells, enriched and decreased CD4+ T cells, enriched and decreased CD8+ T cells, enriched macrophages, enriched and decreased NK T cells, decreased Tregs, and decreased Th1 cell cohorts (Figure 10A–G,I,J,L,N). There was no remarkable correlation between MRPL12 and OS in decreased macrophages, enriched Tregs, enriched Th1 cells, or enriched or decreased Th2 cell cohorts (Figure 10H,K,M,O,P). These data suggest that MRPL12 expression influences the survival of LUAD patients through affecting the infiltration levels of immune cells, including enriched macrophages, decreased Tregs, and decreased Th1 cell cohorts.

### 2.11. MRPL12 Knockdown Inhibited Proliferation and Invasion of LUAD Cells

**Figure 11:**
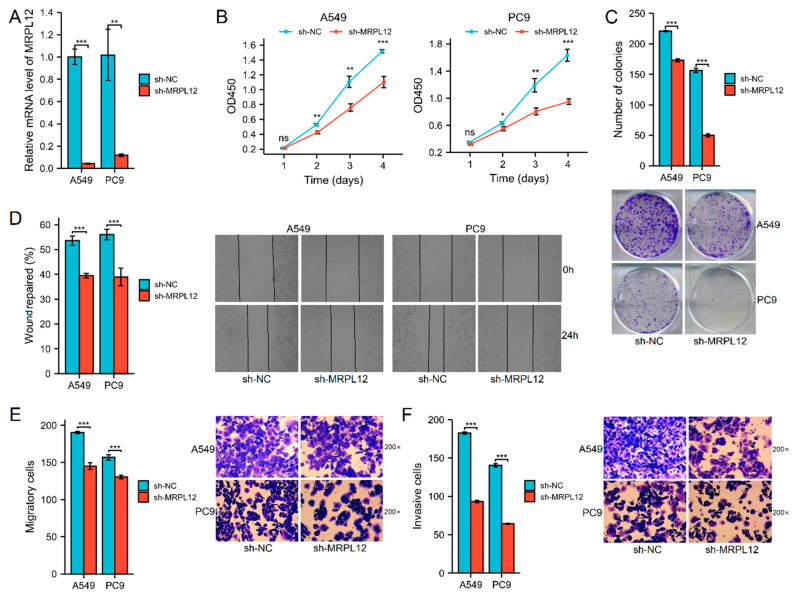
MRPL12 knockdown suppressed cell proliferation and invasion in LUAD. (**A**) MRPL12 mRNA expression in A549 and PC9 cells after sh-MRPL12 transfection. (**B**) Sh-MRPL12 inhibited LUAD cell growth according to CCK-8. (**C**) Sh-MRPL12 inhibited colony number of LUAD cells according to the colony formation assay. (**D**) Sh-MRPL12 reduced wound closure percent of LUAD cells according to wound-healing assay. (**E**,**F**) Sh-MRPL12 suppressed cell migration and invasion in LUAD according to Transwell assays. * *p* < 0.05, ** *p* < 0.01, *** *p* < 0.001; ns, no significance.

Furthermore, to evaluate the functions of MRPL12 in LUAD cell proliferation, migration, and invasion, various cellular experiments were carried out. MRPL12 mRNA expression was significantly decreased in A549 and PC9 cells after sh-MRPL12 transfection (Figure 11A). The CCK-8 assay and colony formation assay indicated MRPL12 knockdown markedly inhibited the proliferation and colony number of LUAD cells (Figure 11B,C). A wound-healing assay revealed that sh-MRPL12 notably reduced the wound closure rate of LUAD cells compared with sh-NC (Figure 11D). The migration and invasion of LUAD cells were dramatically suppressed by sh-MRPL12 (Figure 11E,F). These data demonstrate that MRPL12 knockdown inhibits growth and metastasis in LUAD cells.

## 3. Discussion

MRPL12 has not been widely studied. However, several studies reported that MRPs could function as diagnostic and prognostic biomarkers in several cancers. For instance, higher MRPL13 expression was significantly associated with worse survival, and was identified as a novel prognostic biomarker in breast cancer [16]. Additionally, MRPL35 was remarkably overexpressed in colorectal cancer, while higher MRPL35 expression was correlated with a shorter OS in these patients [17]. The functional role of MRPL12 in LUAD remains unclear.

Comprehensive bioinformatics analyses were carried out to systematically investigate the expression and clinical importance of MRPL12 in LUAD. MRPL12 expression was significantly upregulated in LUAD tissues, and high MRPL12 expression was related to a shorter OS in LUAD patients. Furthermore, MRPL12 was strongly correlated with immune infiltrates, immunomodulators, and chemokines in LUAD. Finally, various cellular experiments demonstrated that MRPL12 knockdown markedly inhibited the proliferation, migration, and invasion of LUAD cells. This research provides new insight into the function of MRPL12, which could act as a prognostic marker linked to immune cell infiltration and tumor development in LUAD.

To investigate the biological functions of MRPL12 in LUAD, co-expressed genes and related pathways of MRPL12 were identified. Further KEGG pathway analysis indicated MRPL12 co-expressed genes participated in ribosomes, oxidative phosphorylation, proteasomes, and the JAK-STAT signaling pathway. Ma et al. demonstrated that MRPL12 positively regulated the mitochondrial oxidative phosphorylation and mitochondrial DNA copy number [18]. Additionally, increasing evidence reveals that mitochondrial oxidative phosphorylation is upregulated in certain cancers, and could be used as an important target for oncotherapy [19]. Chen et al. reported that a novel oxidative phosphorylation inhibitor (IACS-010759) might be beneficial for PD-1-resistant NSCLC and increase anti-tumor immunity [20]. Numerous studies showed that the JAK-STAT signaling pathway plays a critical role in the initiation and development of lung cancer [21,22]. Moreover, MRPL12 has been correlated with several immune-associated biological processes, such as Th1 and Th2 cell differentiation and Th17 cell differentiation. These findings suggest MRPL12 may be crucial in tumor progression through regulating the oxidative phosphorylation function and immune response of LUAD.

The TME is a complex microenvironment composed of diverse cell types, such as malignant cells, immune cells, stromal cells, and endothelial cells. Recently, single-cell RNA sequencing has emerged as a powerful tool for profiling the highly complex TME at the single-cell level [23]. The distribution of MRPL12 expression in various cell types was evaluated using the TISCH database. We found MRPL12 was mainly expressed in malignant cells, endothelial cells, and immune cells, including CD4Tconv cells and CD8T cells. In addition, MRPL12 expression varied widely in different cell types, which might lead to TME heterogeneity in LUAD. As we know, TME components vary considerably among different cancers, and play a critical role in the initiation, progression, and metastasis of tumors [24]. 

TIICs, as important components of the TME, are essential in tumor development, and the infiltration levels of these cells influence the survival outcomes of patients with cancer [25]. In this study, MRPL12 expression was negatively correlated with the infiltration levels of most TIICs, including CD8+ T cells, CD4+ T cells, DCs, NK cells, Th1 cells, and Tregs. Among them, DCs play a crucial role in the initiation of the anti-cancer immune response because they can detect tumor antigens produced by malignant cells, such as mutated or abnormally expressed proteins [26]. Additionally, CD8+ T cells are considered one of primary effector cells in anti-cancer immunity, and the infiltration of CD8+ T cells in the TME is linked to better survival in multiple cancers [27]. CD4+ T cells could regulate anti-cancer immunity either directly by eliminating malignant cells or indirectly by activating innate immune cells [28]. NK cells are immune cells that play a pivotal role in the pathogenesis of lung cancer; the infiltration degree of NK cells was positively correlated with the survival of lung cancer patients [29]. The negative correlation between MRPL12 and these immune cells further supports that MRPL12 overexpression restrains cancer immunity, thus contributing to cancer development and the poor survival of LUAD patients.

The relationship between MRPL12 expression and chemokines or chemokine receptors in LUAD was then analyzed. Chemokines are chemotactic cytokines that can mediate immune cell trafficking [30]. In this research, MRPL12 expression was negatively correlated with many chemokines, including CCL14, CCL19, CCL23, CX3CL1, CXCL12, and CXCL16. Meanwhile, MRPL12 expression was significantly correlated with various chemokine receptors, including CCR2, CCR4, CCR6, CCR7, CX3CR1, and CXCR2. Chemokines and chemokine receptors are critical in the composition of the TME and strongly impact both pro- and anti-tumorigenic immune responses [31]. For example, CCL19-producing fibroblastic stromal cells could suppress tumor growth in NSCLC through facilitating the local anti-tumor T-cell response [32]. Moreover, LUAD patients with high CCR7 or CCL19 mRNA expression had better survival rates than patients with low CCR7 or CCL19 mRNA expression, indicating that CCR7 and CCL19 are good prognostic factors in LUAD [33]. In addition to chemokines, a number of immunomodulators are significantly correlated with MRPL12 expression. These data support that MRPL12 can participate in the immune regulation in LUAD.

The biological function of MRPL12 in LUAD was verified using various cellular experiments. MRPL12 knockdown markedly inhibited cell proliferation, migration, and invasion in LUAD. Similar to our results, Liu et al. demonstrated that MRPL12 overexpression was related to worse survival rates in patients with breast cancer, while MRPL12 knockdown notably inhibited the proliferation and migration of breast cancer cells [11]. These results provide insight into the function of MRPL12 in LUAD progression.

However, several limitations should be mentioned. Firstly, the expression level and biological function of MRPL12 in LUAD cells were validated, but the knockdown efficiency of sh-MRPL12 in LUAD cells was not confirmed by detecting protein expression, and the potential molecular mechanisms of MRPL12 in tumor progression were not examined. Secondly, an animal study is needed to validate the function of MRPL12 in vivo. Thirdly, bioinformatic analyses identified the significant relationship between MRPL12 and immune infiltrates as well as chemokines, which has not been verified in vitro. In a future study, we will investigate the molecular mechanisms and immunoregulatory functions of MRPL12 in LUAD.

## 4. Materials and Methods 

### 4.1. UALCAN Analysis

UALCAN (http://ualcan.path.uab.edu/ (accessed on 10 October 2022)) is web tool used to analyze cancer OMICS data (TCGA, MET500, CPTAC, and CBTTC) [34]. MRPL12 mRNA and protein expression in LUAD and normal lung samples were compared using this database.

### 4.2. HPA Analysis

MRPL12 protein expression was assessed via the IHC method. IHC images of MRPL12 in tumor and normal tissues were randomly downloaded from the HPA database (http://www.proteinatlas.org/ (accessed on 21 October 2022)).

### 4.3. TMA Analysis

A TMA containing 80 pairs of LUAD and matched normal tissues was purchased from Shanghai Outdo Biotech Co., Ltd. (Shanghai, China). IHC was conducted as previously described [35]. The TMA section was incubated with a primary antibody against MRPL12 (1:300, ab154961, Abcam, Cambridge, UK). 

The IHC score was determined from staining intensity and positive cell percentage. Staining intensity was defined as follows: 0 (no), 1 (weak), 2 (moderate), and 3 (strong). Positive cell percentage was defined as follows: 0 (<10%), 1 (11–25%), 2 (26–50%), 3 (51–75%), and 4 (76–100%). Total IHC score was calculated by multiplying staining intensity score by positive score. Samples with an IHC score of ≥6 were categorized into the high-MRPL12-expression cohort, whereas those with an IHC score of <6 were categorized into the low-MRPL12-expression cohort [36].

### 4.4. TIMER Analysis

TIMER (https://cistrome.shinyapps.io/timer/ (accessed on 5 August 2022)) is a comprehensive database used for systematic analyses of immune infiltration in various cancers [37]. We investigated MRPL12 expression in tumor and normal tissues across different TCGA tumors. The relationship between MRPL12 expression and the abundance of 6 immune infiltrates was then explored. Additionally, the correlation between MRPL12 expression and related gene markers of TIICs was also evaluated using TIMER. 

### 4.5. GEPIA Analysis

GEPIA (http://gepia.cancer-pku.cn/index.html (accessed on 12 August 2022)) is a database for analyzing the gene expression data from TCGA [38]. We utilized GEPIA to evaluate the prognostic value of MRPL12 in LUAD patients. GEPIA was then applied to validate the correlations between MRPL12 expression and gene markers of immune cells identified using TIMER.

### 4.6. Kaplan–Meier Plotter Analysis

Kaplan–Meier plotter is an online database (https://kmplot.com/analysis/ (accessed on 3 September 2022)) for analyzing the prognostic value of different genes among 21 cancer types. Survival analysis was carried out on the predictive significance of MRPL12 expression in LUAD. Moreover, the prognostic value of MRPL12 according to immune cells was assessed using this database. 

### 4.7. LinkedOmics Analysis

LinkedOmics (http://www.linkedomics.org/login.php (accessed on 23 October 2022)) is a web tool for analyzing multi-omics data from 32 TCGA cancers and 10 CPTAC cohorts [39]. Genes co-expressed with MRPL12 were screened using the LinkFinder module of this database. Further GO and KEGG pathway enrichment analyses were carried out using the LinkInterpreter module of this database.

### 4.8. PPI Network Analysis

The GeneMANIA database (http://www.genemania.org (accessed on 9 September 2022)) is a web tool used to generate hypotheses regarding gene functions, analyze gene lists, and prioritize genes for functional assays [40]. The PPI network of MRPL12 was explored using GeneMANIA.

### 4.9. TISIDB Analysis

TISIDB (http://cis.hku.hk/TISIDB/index.php (accessed on 10 October 2022)) is a database that integrates many heterogeneous data sources to assess the interactions between tumors and the immune system [41]. To confirm whether MRPL12 is involved in immune infiltration in LUAD, we explored the relationship between MRPL12 and the abundance of TILs, immunomodulators, and chemokines in TISIDB. 

### 4.10. TISCH Analysis

TISCH (http://tisch.comp-genomics.org/ (accessed on 15 October 2022)) is a single-cell RNA sequencing database focusing on the TME [42], which can provide detailed cell-type annotation at the single-cell level, enabling exploration of the TME across a diverse range of cancers. We utilized datasets from this database to analyze MRPL12 expression in the TME as a single-cell subset.

### 4.11. Cell Transfection

Human LUAD cell lines (A549 and PC9) were purchased from the Cell Bank of the Chinese Academy of Sciences (Shanghai, China). They were cultured in RPMI 1640 medium containing 10% FBS (HyClone, Logan, UT, USA). A lentiviral-vector-mediated short hairpin RNA (shRNA) targeting MRPL12 (5′-CAGCCTCACTCTCTTGGAAAT-3′) and a negative control (5′-TTCTCCGAACGTGTCACGT-3′) were synthesized by the GeneChem Corporation (Shanghai, China). They were transfected into cell lines using Lipofectamine 2000 reagent (Invitrogen, Carlsbad, CA, USA).

### 4.12. Quantitative Real-Time PCR Assay

Total RNAs were extracted from cells with the TRIzol reagent (Invitrogen, Carlsbad, CA, USA) and then reverse-transcribed into cDNA using an M-MLV Reverse Transcription Kit (Promega, Madison, WI, USA). A PCR assay was performed using an SYBR Master Mix Kit (Takara, Shiga, Japan) on a LightCycler 480 II real-time PCR instrument (Roche, Basel, Switzerland). Primer sequences: MRPL12 forward, 5′-CTTGTGCCGATGGGTGGT-3′, reverse, 5′-TCAGGCGGACGGTGAAAT-3′. MRPL12 mRNA expression was calculated via the 2^−ΔΔCT^ method.

### 4.13. Cell Proliferation Assay

A CCK-8 assay was utilized to detect cell proliferation. Transfected cells were seeded into 96-well plates, and then cultured for 1, 2, 3, or 4 days. CCK-8 reagent (Dojindo, Shanghai, China) was added into each well. The absorbance at 450 nm was examined with a microplate reader (Thermo Fisher Scientific, Waltham, MA, USA).

### 4.14. Colony Formation Assay

Transfected cells were seeded into 6-well plates, and then cultured in RPMI 1640 medium supplemented with 10% FBS. Culture was terminated when colonies were visible. Cells were fixed in 4% paraformaldehyde, and stained with 0.5% crystal violet. The colony number was counted under a fluorescence microscope (Olympus, Tokyo, Japan).

### 4.15. Wound-Healing Assay

Transfected cells were seeded into 6-well plates, and then a scratch wound was made using the pipette tip. The dish was washed with PBS to remove detached cells, and cells were cultured in a low-concentration serum medium. Photographs were collected at 0 and 24 h, while the wound closure rate was analyzed.

### 4.16. Transwell Assay

A Transwell chamber (Corning, NY, USA) was utilized to examine cell migration and invasion. For cell migration, 100 µL of serum-free RPMI 1640 medium (containing 1 × 10^5^ cells) was added to the upper chamber, whereas 600 µL of RPMI 1640 medium supplemented with 20% FBS without cells was added to the lower chamber. After 24 h, cells on the lower surface of membrane were fixed with 4% paraformaldehyde, stained with 0.5% crystal violet, and counted. For cell invasion, the upper chamber was pre-coated with Matrigel (BD Biosciences, San Diego, CA, USA), and subsequent procedures were the same as above.

### 4.17. Statistical Analysis

Statistical analyses were performed using online bioinformatics databases and SPSS 22.0 software (IBM, Armonk, NY, USA). A Kaplan–Meier curve was applied to evaluate the relationship between MRPL12 expression and OS. Associations between MRPL12 expression and immune infiltrates, immunomodulators, and chemokines were assessed via Spearman correlation analysis. To identify prognostic factors, univariate and multivariate Cox regression analyses were carried out. Experimental data were presented as mean ± SD, and compared using Student’s *t*-test. Multiple group comparisons were carried out with one-way ANOVA, followed by Bonferroni post hoc test. Each assay was replicated in at least 3 independent experiments. Statistical significance was set at *p* < 0.05.

## 5. Conclusions

MRPL12 expression was remarkably higher in LUAD tissues, and was correlated with unfavorable clinicopathological factors and worse survival in LUAD patients. Moreover, high MRPL12 expression was significantly associated with low infiltration levels of multiple immune cells. Cellular functional experiments revealed that MRPL12 knockdown markedly inhibited the proliferation, migration, and invasion of LUAD cells. These findings suggest that MRPL12 is a novel prognostic biomarker as well as a potential therapeutic target for LUAD.

## Figures and Tables

**Table 1:** Correlation between MRPL12 expression and clinicopathological information in LUAD patients from TMA.

Variables	N	MRPL12 Expression		*p* Value
		Low (n = 31)	High (n = 49)	
Age (years)				0.910
≤60	29	11	18	
>60	51	20	31	
Gender				0.050
Female	33	17	16	
Male	47	14	33	
Tumor size (cm)				0.185
≤3	39	18	21	
>3	41	13	28	
T stage				0.102
T1/T2	59	26	33	
T3/T4	21	5	16	
N stage				0.433
N0	58	24	34	
N1/N2	22	7	15	
Pathologic stage				0.003
I/II	51	26	25	
III/IV	29	5	24	
Chemotherapy				0.009
No	42	22	20	
Yes	38	9	29	
Survival status				0.001
Alive	49	26	23	
Dead	31	5	26	
Survival time (months)		68.22 ± 22.02	43.56 ± 23.21	<0.001

**Table 2:** Univariate and multivariate Cox regression analyses in LUAD patients from TMA.

Variables	Univariate Analysis			Multivariate Analysis		
	OR	95%CI	*p*	OR	95%CI	*p*
Age (years)						
≤60	ref					
>60	0.703	0.344–1.437	0.334			
Gender						
Female	ref					
Male	1.576	0.737–3.369	0.240			
Tumor size (cm)						
≤3	ref			ref		
>3	3.677	1.631–8.292	0.001	0.72	0.289–1.793	0.481
T stage						
T1/T2	ref			ref		
T3/T4	13.87	6.139–31.32	<0.001	5.455	1.713–17.368	0.004
N stage						
N0	ref			ref		
N1/N2	3.315	1.633–6.728	<0.001	1.144	0.418–3.131	0.792
Pathologic stage						
I/II	ref			ref		
III/IV	32.17	10.95–94.47	<0.001	9.477	1.537–58.42	0.015
Chemotherapy						
No	ref			ref		
Yes	12.85	4.47–36.96	<0.001	2.685	0.687–10.485	0.155
MRPL12 expression						
Low	ref			ref		
High	5.611	1.942–16.21	0.001	3.861	1.187–12.562	0.024

**Table 3:** Correlation between MRPL12 and related gene markers of immune cells in TIMER.

Description	Gene Markers	None Cor	*p* Value	Purity Cor	*p* Value
CD8+ T cell	CD8A	−0.058	0.187	−0.016	0.718
	CD8B	0.007	0.872	0.044	0.325
T cell (general)	CD3D	−0.121	**	−0.077	0.087
	CD3E	−0.222	***	−0.196	***
	CD2	−0.224	***	−0.2	***
B cell	CD19	−0.194	***	−0.165	***
	CD79A	−0.201	***	−0.172	***
Monocyte	CD86	−0.213	***	−0.191	***
	CD115 (CSF1R)	−0.272	***	−0.251	***
TAM	CCL2	−0.126	***	−0.096	*
	CD68	−0.191	***	−0.169	***
	IL10	−0.181	***	−0.151	***
M1 macrophage	INOS (NOS2)	0.002	0.964	0.009	0.835
	IRF5	−0.067	0.13	−0.033	0.467
	COX2(PTGS2)	−0.026	0.562	−0.039	0.392
M2 macrophage	CD163	−0.209	***	−0.191	***
	VSIG4	−0.193	***	−0.175	***
	MS4A4A	−0.25	***	−0.23	***
Neutrophils	CD66b (CEACAM8)	−0.35	***	−0.35	***
	CD11b (ITGAM)	−0.305	***	−0.281	***
	CCR7	−0.308	***	−0.287	***
NK cell	KIR2DL1	0	0.993	0.012	0.785
	KIR2DL3	−0.002	0.97	0.022	0.629
	KIR2DL4	0.185	***	0.21	***
	KIR3DL1	−0.031	0.481	−0.013	0.772
	KIR3DL2	0.017	0.705	0.053	0.245
	KIR3DL3	0.068	0.123	0.082	0.068
	KIR2DS4	−0.063	0.152	−0.038	0.397
DC	HLA-DPB1	−0.409	***	−0.401	***
	HLA-DQB1	−0.258	***	−0.24	***
	HLA-DRA	−0.37	***	−0.362	***
	HLA-DPA1	−0.408	***	−0.398	***
	BCDA-1 (CD1C)	−0.439	***	−0.42	***
	BDCA-4 (NRP1)	−0.144	**	−0.136	**
	CD11c (ITGAX)	−0.238	***	−0.218	***
Th1 cell	T-bet (TBX21)	−0.158	***	−0.133	**
	STAT4	−0.21	***	−0.196	***
	STAT1	0.029	0.506	0.049	0.278
	IFN-γ (IFNG)	0.074	0.093	0.114	*
	TNF-α (TNF)	−0.162	***	−0.122	**
Th2 cell	GATA3	−0.198	***	−0.169	***
	STAT6	−0.302	***	−0.326	***
	STAT5A	−0.288	***	−0.263	***
	IL13	−0.065	0.139	−0.051	0.257
Th17 cell	STAT3	−0.242	***	−0.256	***
	IL17A	0.022	0.617	0.048	0.289
Treg	FOXP3	−0.115	**	−0.086	0.057
	CCR8	−0.219	***	−0.196	***
	STAT5B	−0.241	***	−0.233	***
	TGFβ (TGFB1)	−0.201	***	−0.178	***
T-cell exhaustion	PD-1 (PDCD1)	0.042	0.341	0.101	*
	PDL1 (PDCD1LG2)	−0.148	***	−0.11	*
	CTLA4	−0.114	**	−0.076	0.093
	LAG3	0.047	0.283	0.09	*
	TIM-3 (HAVCR2)	−0.189	***	−0.164	***
	GZMB	0.176	***	0.232	***

* *p* < 0.05, ** *p* < 0.01, *** *p* < 0.001.

**Table 4:** Correlation between MRPL12 and related gene markers of immune cells in GEPIA.

Description	Gene Markers	Tumor Cor	*p* Value	Normal Cor	*p* Value
T cell (general)	CD3D	−0.17	***	0.13	0.31
	CD3E	−0.28	***	−0.11	0.4
	CD2	−0.28	***	−0.044	0.74
B cell	CD19	−0.29	***	0.07	0.6
	CD79A	−0.32	***	−0.1	0.45
TAM	CCL2	−0.13	**	−0.26	*
	CD68	−0.2	***	0.042	0.75
	IL10	−0.23	***	−0.029	0.83
M2 macrophage	CD163	−0.16	***	0.13	0.32
	VSIG4	−0.2	***	0.17	0.19
	MS4A4A	−0.27	***	0.16	0.23
DC	HLA-DPB1	−0.39	***	0.12	0.35
	HLA-DQB1	−0.18	***	−0.044	0.74
	HLA-DRA	−0.36	***	0.21	0.11
	HLA-DPA1	−0.4	***	−0.22	0.096
	BCDA-1 (CD1C)	−0.37	***	0.083	0.53
	BDCA-4 (NRP1)	−0.12	**	−0.44	***
	CD11c (ITGAX)	−0.26	***	−0.017	0.9
Th1 cell	T-bet (TBX21)	−0.23	***	−0.1	0.44
	STAT4	−0.21	***	−0.27	*
	STAT1	−0.009	0.85	−0.19	0.14
	IFN-γ (IFNG)	−0.008	0.86	0.03	0.82
	TNF-α (TNF)	−0.18	***	−0.034	0.8
Th2 cell	GATA3	−0.21	***	−0.14	0.31
	STAT6	−0.24	***	−0.37	**
	STAT5A	−0.3	***	−0.067	0.61
	IL13	−0.098	*	−0.16	0.22
Treg	FOXP3	−0.15	**	0.029	0.83
	CCR8	−0.26	***	−0.12	0.36
	STAT5B	−0.22	***	−0.53	***
	TGFβ (TGFB1)	−0.15	***	0.14	0.3

* *p* < 0.05, ** *p* < 0.01, *** *p* < 0.001.

## Data Availability

Publicly available datasets were analyzed in this study. The data can be found here: UALCAN, http://ualcan.path.uab.edu/ (accessed on 10 October 2022); HPA, http://www.proteinatlas.org/ (accessed on 21 October 2022); TIMER, https://cistrome.shinyapps.io/timer/ (accessed on 5 August 2022); GEPIA, http://gepia.cancer-pku.cn/index.html (accessed on 12 August 2022); Kaplan–Meier plotter, https://kmplot.com/analysis/ (accessed on 3 September 2022); LinkedOmics, http://www.linkedomics.org/login.php (accessed on 23 October 2022); GeneMANIA, http://www.genemania.org (accessed on 9 September 2022); TISIDB http://cis.hku.hk/TISIDB/index.php (accessed on 10 October 2022); and TISCH, http://tisch.comp-genomics.org/ (accessed on 15 October 2022).

## References

[1] Sung H., Ferlay J., Siegel R.L., Laversanne M., Soerjomataram I., Jemal A., Bray F. (2021). Global Cancer Statistics 2020: GLOBOCAN Estimates of Incidence and Mortality Worldwide for 36 Cancers in 185 Countries. CA Cancer J. Clin..

[2] Chen Z., Fillmore C.M., Hammerman P.S., Kim C.F., Wong K.K. (2014). Non-small-cell lung cancers: A heterogeneous set of diseases. Nat. Rev. Cancer.

[3] Denisenko T.V., Budkevich I.N., Zhivotovsky B. (2018). Cell death-based treatment of lung adenocarcinoma. Cell Death Dis..

[4] Allemani C., Matsuda T., Di Carlo V., Harewood R., Matz M., Niksic M., Bonaventure A., Valkov M., Johnson C.J., Estève J. (2018). Global surveillance of trends in cancer survival 2000-14 (CONCORD-3): Analysis of individual records for 37,513,025 patients diagnosed with one of 18 cancers from 322 population-based registries in 71 countries. Lancet.

[5] Cheong A., Lingutla R., Mager J. (2020). Expression analysis of mammalian mitochondrial ribosomal protein genes. Gene Expr. Patterns.

[6] Surovtseva Y.V., Shutt T.E., Cotney J., Cimen H., Chen S.Y., Koc E.C., Shadel G.S. (2011). Mitochondrial ribosomal protein L12 selectively associates with human mitochondrial RNA polymerase to activate transcription. Proc. Natl. Acad. Sci. USA.

[7] Kim H.J., Maiti P., Barrientos A. (2017). Mitochondrial ribosomes in cancer. Semin. Cancer Biol..

[8] Min S., Lee Y.K., Hong J., Park T.J., Woo H.G., Kwon S.M., Yoon G. (2021). MRPS31 loss is a key driver of mitochondrial deregulation and hepatocellular carcinoma aggressiveness. Cell Death Dis..

[9] Gu X., Liu Y., Wang N., Zhen J., Zhang B., Hou S., Cui Z., Wan Q., Feng H. (2021). Transcription of MRPL12 regulated by Nrf2 contributes to the mitochondrial dysfunction in diabetic kidney disease. Free Radic. Biol. Med..

[10] Chen Y., Cairns R., Papandreou I., Koong A., Denko N.C. (2009). Oxygen consumption can regulate the growth of tumors, a new perspective on the Warburg effect. PLoS ONE.

[11] Liu Y., Sun H., Li X., Liu Q., Zhao Y., Li L., Xu B., Hou Y., Jin W. (2021). Identification of a Three-RNA Binding Proteins (RBPs) Signature Predicting Prognosis for Breast Cancer. Front. Oncol..

[12] Ye L., Zhang T., Kang Z., Guo G., Sun Y., Lin K., Huang Q., Shi X., Ni Z., Ding N. (2019). Tumor-Infiltrating Immune Cells Act as a Marker for Prognosis in Colorectal Cancer. Front. Immunol..

[13] Wu S., Yang W., Zhang H., Ren Y., Fang Z., Yuan C., Yao Z. (2019). The Prognostic Landscape of Tumor-Infiltrating Immune Cells and Immune Checkpoints in Glioblastoma. Technol. Cancer Res. Treat..

[14] Liu X., Shang X., Li J., Zhang S. (2021). The Prognosis and Immune Checkpoint Blockade Efficacy Prediction of Tumor-Infiltrating Immune Cells in Lung Cancer. Front. Cell Dev. Biol..

[15] Zeng Y., Shi Y., Xu L., Zeng Y., Cui X., Wang Y., Yang N., Zhou F., Zhou Y. (2021). Prognostic Value and Related Regulatory Networks of MRPL15 in Non-Small-Cell Lung Cancer. Front. Oncol..

[16] Ye H., Zhang N. (2021). Identification of the Upregulation of MRPL13 as a Novel Prognostic Marker Associated with Overall Survival Time and Immunotherapy Response in Breast Cancer. Comput. Math. Methods Med..

[17] Zhang L., Lu P., Yan L., Yang L., Wang Y., Chen J., Dai J., Li Y., Kang Z., Bai T. (2019). MRPL35 Is Up-Regulated in Colorectal Cancer and Regulates Colorectal Cancer Cell Growth and Apoptosis. Am. J. Pathol..

[18] Ma Y., Zhu S., Lv T., Gu X., Feng H., Zhen J., Xin W., Wan Q. (2020). SQSTM1/p62 Controls mtDNA Expression and Participates in Mitochondrial Energetic Adaption via MRPL12. iScience.

[19] Ashton T.M., McKenna W.G., Kunz-Schughart L.A., Higgins G.S. (2018). Oxidative Phosphorylation as an Emerging Target in Cancer Therapy. Clin. Cancer Res..

[20] Chen D., Barsoumian H.B., Fischer G., Yang L., Verma V., Younes A.I., Hu Y., Masropour F., Klein K., Vellano C. (2020). Combination treatment with radiotherapy and a novel oxidative phosphorylation inhibitor overcomes PD-1 resistance and enhances antitumor immunity. J. Immunother. Cancer.

[21] Gao Y., Luo L., Xie Y., Zhao Y., Yao J., Liu X. (2020). PYCR1 knockdown inhibits the proliferation, migration, and invasion by affecting JAK/STAT signaling pathway in lung adenocarcinoma. Mol. Carcinog..

[22] Prabhu K.S., Bhat A.A., Siveen K.S., Kuttikrishnan S., Raza S.S., Raheed T., Jochebeth A., Khan A.Q., Chawdhery M., Haris M. (2021). Sanguinarine mediated apoptosis in Non-Small Cell Lung Cancer via generation of reactive oxygen species and suppression of JAK/STAT pathway. Biomed. Pharmacother..

[23] Potter S.S. (2018). Single-cell RNA sequencing for the study of development, physiology and disease. Nat. Rev. Nephrol..

[24] He D., Wang D., Lu P., Yang N., Xue Z., Zhu X., Zhang P., Fan G. (2021). Single-cell RNA sequencing reveals heterogeneous tumor and immune cell populations in early-stage lung adenocarcinomas harboring EGFR mutations. Oncogene.

[25] Wang Y., Yin C., Geng L., Cai W. (2020). Immune Infiltration Landscape in Clear Cell Renal Cell Carcinoma Implications. Front. Oncol..

[26] de Winde C.M., Munday C., Acton S.E. (2020). Molecular mechanisms of dendritic cell migration in immunity and cancer. Med. Microbiol. Immunol..

[27] Fu C., Jiang A. (2018). Dendritic Cells and CD8 T Cell Immunity in Tumor Microenvironment. Front. Immunol..

[28] Kravtsov D.S., Erbe A.K., Sondel P.M., Rakhmilevich A.L. (2022). Roles of CD4+ T cells as mediators of antitumor immunity. Front. Immunol..

[29] Aktas O.N., Ozturk A.B., Erman B., Erus S., Tanju S., Dilege S. (2018). Role of natural killer cells in lung cancer. J. Cancer Res. Clin. Oncol..

[30] Nagarsheth N., Wicha M.S., Zou W. (2017). Chemokines in the cancer microenvironment and their relevance in cancer immunotherapy. Nat. Rev. Immunol..

[31] Ozga A.J., Chow M.T., Luster A.D. (2021). Chemokines and the immune response to cancer. Immunity.

[32] Cheng H.W., Onder L., Cupovic J., Boesch M., Novkovic M., Pikor N., Tarantino I., Rodriguez R., Schneider T., Jochum W. (2018). CCL19-producing fibroblastic stromal cells restrain lung carcinoma growth by promoting local antitumor T-cell responses. J. Allergy Clin. Immunol..

[33] Itakura M., Terashima Y., Shingyoji M., Yokoi S., Ohira M., Kageyama H., Matui Y., Yoshida Y., Ashinuma H., Moriya Y. (2013). High CC chemokine receptor 7 expression improves postoperative prognosis of lung adenocarcinoma patients. Br. J. Cancer.

[34] Chandrashekar D.S., Bashel B., Balasubramanya S.A.H., Creighton C.J., Ponce-Rodriguez I., Chakravarthi B., Varambally S. (2017). UALCAN: A Portal for Facilitating Tumor Subgroup Gene Expression and Survival Analyses. Neoplasia.

[35] Zhang C., Wang C., Yang Z., Bai Y., Shukuya T., Poh M.E., Ekman S., Li J., Xu Y., Deng S. (2022). Identification of GPX4 as a therapeutic target for lung adenocarcinoma after EGFR-TKI resistance. Transl. Lung Cancer Res..

[36] Lei Y., Yu T., Li C., Li J., Liang Y., Wang X., Chen Y., Wang X. (2021). Expression of CAMK1 and its association with clinicopathologic characteristics in pancreatic cancer. J. Cell. Mol. Med..

[37] Li T., Fan J., Wang B., Traugh N., Chen Q., Liu J.S., Li B., Liu X.S. (2017). TIMER: A Web Server for Comprehensive Analysis of Tumor-Infiltrating Immune Cells. Cancer Res..

[38] Tang Z., Li C., Kang B., Gao G., Li C., Zhang Z. (2017). GEPIA: A web server for cancer and normal gene expression profiling and interactive analyses. Nucleic Acids Res..

[39] Vasaikar S.V., Straub P., Wang J., Zhang B. (2018). LinkedOmics: Analyzing multi-omics data within and across 32 cancer types. Nucleic Acids Res..

[40] Warde-Farley D., Donaldson S.L., Comes O., Zuberi K., Badrawi R., Chao P., Franz M., Grouios C., Kazi F., Lopes C.T. (2010). The GeneMANIA prediction server: Biological network integration for gene prioritization and predicting gene function. Nucleic Acids Res..

[41] Ru B., Wong C.N., Tong Y., Zhong J.Y., Zhong S.S.W., Wu W.C., Chu K.C., Wong C.Y., Lau C.Y., Chen I. (2019). TISIDB: An integrated repository portal for tumor-immune system interactions. Bioinformatics.

[42] Sun D., Wang J., Han Y., Dong X., Ge J., Zheng R., Shi X., Wang B., Li Z., Ren P. (2021). TISCH: A comprehensive web resource enabling interactive single-cell transcriptome visualization of tumor microenvironment. Nucleic Acids Res..

